# The Relationship Between Clinical Nurses’ Perceptions of the Power Used by Nursing Managers and Professional Silence Behaviors: An Example From Türkiye

**DOI:** 10.1155/jonm/6581986

**Published:** 2026-03-17

**Authors:** Şirin Çelikkanat, Ayşe Eminoğlu Güven, Zeynep Güngörmüş

**Affiliations:** ^1^ Gaziantep Islam Science and Technology University of Health Sciences Department of Nursing, Gaziantep, Turkey

**Keywords:** employee silence, nurse manager, nursing, power source

## Abstract

**Background:**

The power that nurse managers exercise significantly determines the extent to which clinical nurses can voice their opinions and communicate effectively within the organization.

**Aim:**

This study aimed to examine the relationships between clinical nurses’ perceptions of nurse managers’ use of power and nurses’ professional silence.

**Method:**

This cross‐sectional study was conducted with 292 clinical nurses from July 25, 2024, to February 17, 2025. Data were collected using a personal information form (comprising the Nurse Manager Perception Questionnaire [NMPQ] in its second section), the Employee Silence Behavior Scale, and the Perceived Power in Nurse Managers Scale. Data were analyzed using the Mann–Whitney *U* test and Pearson correlation analysis.

**Results:**

The findings revealed that single nurses and those without children perceived nurse managers’ power differently. Clinical nurses who believed that their nurse managers met expectations, communicated effectively, and adopted a team‐oriented approach had significantly higher perceptions of “charismatic power,” “reward power,” “legitimate power,” and “expert power” (*p* < 0.05). Conversely, perceptions of coercive power were higher among clinical nurses who could not communicate effectively with their managers and who did not believe their managers influenced their working conditions (*p* < 0.05). A significant positive correlation was found between clinical nurses’ silence and their perception of nurse managers’ coercive power (*p* < 0.05).

**Conclusion:**

The study concluded that as clinical nurses’ perception of nurse managers’ coercive power increased, their professional silence also increased. Conversely, effective communication, meeting expectations, and a team‐oriented approach enhanced their perceptions of nurse managers’ charismatic, expert, legitimate, and reward power. These findings suggest that how nurse managers use power decisively influences the communication and silence behaviors of clinical nurses. Therefore, organizational and managerial strategies aimed at reducing clinical nurses’ silence are essential for ensuring a safe working environment and delivering quality nursing care.

## 1. Introduction

The health sector is characterized by an intense work pace, critical decision‐making processes, and a hierarchical structure, making effective communication and power balances particularly important. Nursing is a professional discipline in which team communication and employee participation are essential for ensuring the continuity and quality of patient care [1]. However, research indicates that clinical nurses in healthcare organizations frequently refrain from providing feedback to managers or expressing concerns about operational issues [2]. This situation reflects an important phenomenon known as “employee silence.” Nurses’ silence refers to their withholding of thoughts, questions, concerns, or knowledge about important issues that affect the quality of care, patient safety, and their own peace of mind [3]. This situation may diminish clinical nurses’ intention to remain in the profession, create conflict between their family and work life, reduce their professional commitment, trigger bullying in subordinate–superior relationships within the institution, and increase their intention to change jobs [4]. Furthermore, it can lead to significant problems that compromise the delivery of healthcare services and hinder patients and their families from receiving quality nursing care [5, 6].

Silence among clinical nurses is a frequently observed phenomenon [4]. Nurses’ silence is associated with individual, interpersonal, and organizational characteristics in healthcare settings [2]. Among these, interactional factors arise from nurses’ interactions with their colleagues and managers [7]. Employee silence is a social process shaped by interaction [3]. Nurses may remain silent out of concern that expressing their opinions could harm their relationship with their nurse managers or put them at risk. The fear of receiving negative feedback from managers and the desire to avoid confrontational situations often lead employees to remain silent [7]. In this context, managers’ leadership styles and their use of power emerge as key determinants of employee silence [8]. Clinical nurses’ perception of their nurse managers’ power is a factor that can directly affect their willingness to express their opinions, potentially causing them to hesitate or remain silent [9].

The sources of managerial power are typically classified into five categories: coercive, reward, legitimate, expert, and charismatic power [10]. While coercive and legitimate power, which are considered authoritarian in nature, may increase employee silence, positive power types such as expert and charismatic power tend to encourage more open communication among employees [8]. In this context, a key question arises: How do nurse managers’ use of power influence clinical nurses’ silence? When power is used in an oppressive, coercive, or punitive manner, employees may remain silent about reporting errors or suggesting process improvements. Conversely, a supportive and encouraging approach to the use of power can motivate employees to actively voice their opinions and contribute to organizational development [11].

Healthcare institutions are organizations where hierarchical structures and professional roles are clearly defined. How nurses perceive nurse managers’ use of power plays a decisive role in their responses to management and their tendency to remain silent. Although professional silence is recognized in the literature as a phenomenon with critical implications for patient safety, quality of care, and organizational learning, the managerial dynamics underlying this behavior have not been fully explored in the nursing field. Furthermore, while professional silence has been examined in relation to variables such as organizational justice, leadership style, and psychological safety, research on how employees perceive managers’ power sources remains limited. However, the perception of power directly influences employees’ silence behavior by shaping their freedom of expression and risk perception, and it forms the basis of managerial interaction. Therefore, this study aims to make a significant theoretical and practical contribution by examining the concepts of power perception and professional silence together. The goal is to deepen the understanding of managerial processes in clinical nursing settings and to inform interventions aimed at preventing professional silence.

Additionally, most studies have been conducted in Western healthcare systems, while evidence from developing countries remains limited. No research to date has examined the relationship between clinical nurses’ perceptions of nurse managers’ power and their silence in the Turkish healthcare system. With its hierarchical structure, organizational culture, and recent healthcare reforms, the Turkish healthcare system offers a unique context for investigation. This study aims to fill this gap by providing the first empirical evidence from Türkiye and contributing to the international literature on context‐specific management approaches. Given these factors, understanding how clinical nurses perceive nurse managers’ use of power and how this relates to their silence is essential for improving organizational communication and patient safety in Turkish healthcare settings. Thus, this study investigates the relationship between clinical nurses’ perceptions of nurse managers’ power use and their silence. The following research questions guided this study:

- What are clinical nurses’ perceptions of nurse managers’ use of power, and what is their level of silence?
- How do clinical nurses perceive their nurse managers’ use of power, and do their silence behaviors vary based on personal characteristics?
- Is there a relationship between clinical nurses’ perceptions of nurse managers’ use of power and their silence behavior?

## 2. Material and Method

### 2.1. Research Design

This quantitative study employed a cross‐sectional, descriptive, and correlational design.

#### 2.1.1. Population and Sample of the Study

The study population comprised clinical nurses working in public, private, and university hospitals in Gaziantep Province, none of whom held current or previous managerial positions. The sample was drawn from this population. The sample size was calculated using G ∗ Power 3.1 software. A review of previous studies [12] informed the determination of the expected confidence intervals for the Perceived Power in Nurse Managers Scale. Accordingly, the required sample size was calculated with *α* = 0.05, power (1 − *β*) = 0.95, and effect size (*p*) = 0.4034112, resulting in a target of 268 participants. To account for potential data loss, the study was ultimately conducted with 292 participants.

### 2.2. Data Collection

In this study, data were collected via an online survey to allow participants to respond more comfortably and objectively. Given that participants might feel hesitant or pressured if the questionnaire, which included items about nurse managers, was administered face‐to‐face, an online platform ensuring anonymity and encouraging voluntary participation was chosen. This approach was intended to enable clinical nurses to express their opinions more freely. After informing hospital administrators about the purpose and scope of the study and obtaining verbal consent, the questionnaires were shared on nurses’ social media groups. Completing the questionnaire took approximately 5–10 min.

### 2.3. Data Collection Tools

The data of the study were collected with the personal information form (comprising the Nurse Manager Perception Questionnaire [NMPQ] in its second section), the Employee Silence Behavior Scale, and the Perceived Power in Nurse Managers Scale.

#### 2.3.1. Personal Information Form

The form, developed by the researchers based on the relevant literature, consists of two sections. The first section includes questions on participants’ sociodemographic and professional characteristics, such as age, marital status, parental status, years of experience in the profession, and work unit. Work units were categorized as either internal medicine or surgical units. Internal medicine units comprised departments such as neurology, pulmonology, nephrology, and cardiology, whereas surgical units included departments such as urology, orthopedics, and neurosurgery.

#### 2.3.2. NMPQ

The second section of the form assesses clinical nurses’ perceptions of their nurse managers. This section includes items addressing (a) the extent to which nurse managers meet clinical nurses’ expectations, (b) their effective communication skills, (c) their approach to care processes, (d) their influence on working conditions, and (e) their team approach. The items were developed from previous studies and theoretical frameworks on leadership, managerial behaviors, and organizational communication in nursing [12–15] (see Appendix 1).

Prior to data collection, the form was reviewed by experts in nursing management to assess its content validity and clarity. In addition, a pilot study was carried out with a group of clinical nurses who were not part of the main sample. Based on feedback from the pilot study, minor revisions were made to the wording of the items, and the final version of the form was used in the main study.

#### 2.3.3. Employee Silence Behavior Scale

Developed by Alparslan et al., this scale aims to identify the reasons why nurses remain silent about workplace improvements and errors. The scale comprises 26 items across five subdimensions: lack of trust in management, fear of top management’s reaction, getting along with colleagues, tendency for prosocial behavior, and passive personality. This five‐point Likert‐type scale is scored from 1 (*strongly disagree*) to 5 (*strongly agree*). Cronbach’s alpha coefficient for the Employee Silence Behavior Scale was reported as 0.93 [16]. In the present study, Cronbach’s alpha coefficient for the scale was calculated as 0.96.

#### 2.3.4. Perceived Power in Nurse Managers Scale

This scale, developed by Karadaş and Yıldırım, consists of 35 Likert‐type items ranging from 1 to 5, on which participants indicate their level of agreement. The scale is a 5‐point Likert‐type scale and is scored as follows: “*Strongly Agree*” = 5 points, “*Agree*” = 4 points, “*Undecided*” = 3 points, “*Disagree*” = 2 points, and “*Strongly Disagree*” = 1 point. Two items (17 and 18) in the reward power subdimension are reverse‐scored. Since the scale is multidimensional, mean scores are calculated separately for each subdimension. Higher mean scores on a subdimension indicate that nurses perceive their managers as exhibiting that particular power source more strongly. The following scoring ranges were used: 1.00–1.79 = *Strongly Disagree*, 1.80–2.59 = *Disagree*, 2.60–3.39 = *Undecided*, 3.40–4.19 = *Agree*, and 4.20–5.00 = *Strongly Agree*. The scale has five subdimensions: charismatic power (Items 1–7), coercive power (Items 8–13), reward power (Items 14–20), legitimate power (Items 21–28), and expert ower (Items 29–35). Cronbach’s alpha coefficient for the scale was reported as 0.922 [17]. In the present study, Cronbach’s alpha coefficient was 0.93.

### 2.4. Data Analysis

The research data were analyzed using the Statistical Package for the Social Sciences (SPSS) for Windows, Version 27.0. Percentage distributions, means and standard deviations, independent samples *t*‐test, the Mann–Whitney *U* test, and Pearson correlation analysis were used to analyze the data. Statistical significance was set at *p* < 0.05.

### 2.5. Ethical Aspects of the Study

This study was performed in accordance with the principles of the Declaration of Helsinki. Participation was entirely voluntary, and all participants received detailed information about the purpose, scope, data collection procedures, and potential risks. The first question on the questionnaire required participants to provide their consent, thereby ensuring that informed consent was obtained from all respondents. They were also explicitly informed of their right to withdraw from the study at any time. Participants’ identities were kept confidential; all data were anonymized prior to analysis and used solely for scientific purposes. Ethical approval for this noninterventional clinical research was granted on July 2, 2024 (approval number: 450.39.15).

## 3. Results

Table 1 presents the total and subscale scores of the Employee Silence Behavior Scale. Nurses scored above the mean on the lack of trust in management (14.21 ± 5.72) and fear of top management’s reaction (23.17 ± 9.54) subscales and below the mean on the getting along with colleagues (10.34 ± 4.28), tendency for prosocial behavior (10.88 ± 4.07), and passive personality (4.70 ± 1.92) subscales, as well as on the total scale (63.32 ± 22.31).

**TABLE 1:** Distribution of Employee Silence Behavior Scale total and subscale scores (*N*: 292).

Scale	Min score	Max score	Mean ± SD
Reasons for nurses’ remaining silent total	26	130	63.32 ± 22.31
Lack of confidence in senior management	6	30	14.21 ± 5.72
Fear of the senior management’s reaction	10	50	23.17 ± 9.54
Getting along with coworkers	4	20	10.34 ± 4.28
Tendency toward prosocial behavior	4	20	10.88 ± 4.07
Meek personality	2	10	4.70 ± 1.92

*Note:* Min = minimum value, Max = maximum value.

Abbreviation: SD, standard deviation.

Table 2 presents the subscale scores of the Perceived Power of Nurse Managers Scale. Nurses scored above the mean on the charismatic power (24.90 ± 5.48), reward power (24.57 ± 5.28), legitimate power (28.10 ± 6.09), and expert power (24.35 ± 6.93) subscales, whereas they scored below the mean on the coercive power (16.28 ± 4.89) subscale.

**TABLE 2:** Distribution of Perceived Power of Nurse Managers Scale (*N*: 292).

Scale	Min score	Max score	Mean ± SD
Charismatic power	7	35	24.90 ± 5.48
Coercive power	6	30	16.28 ± 4.89
Rewarding power	7	35	24.57 ± 5.28
Legal power	8	40	28.10 ± 6.09
Expertise power	7	35	24.35 ± 6.93

*Note:* Min = minimum value, Max = maximum value.

Abbreviation: SD, standard deviation.

No significant relationship was found between gender or educational status and the scale scores (*p* > 0.05). Single and childless participants had significantly higher scores on the charismatic, expert, and legitimate power subscales (*p* < 0.05). Nurses working in internal medicine units reported significantly higher levels of silence behavior and perceptions of coercive power (*p* < 0.05). Nurses with 1–4 years of experience had significantly higher scores on silence behavior, charismatic power, legitimate power, and expert power compared to those with more than 5 years of experience (*p* < 0.05) (Table 3).

**TABLE 3:** Distribution of participants’ scale scores according to personal characteristics.

Characteristics	N:292	Reasons for nurses’ remaining silent total	Charismatic power	Coercive power	Rewarding power	Legal power	Expertise power
Gender	Male (99)	146.69	139.76	153.60	144.63	143.23	141.85
	Female (193)	146.40	149.96	142.86	147.46	148.18	148.89

Statistics		*p*: 0.978 MWU: 9535.0	*p*: 0.332 MWU: 8886.0	*p*: 0.302 MWU: 8850.5	*p*: 0.785 MWU: 9368.0	*p*: 0.635 MWU: 9230.0	*p*: 0.499 MWU: 9093.0

Marital status	Married (158)	64.68 ± 20.22	24.27 ± 5.10	16.09 ± 4.56	24.26 ± 5.16	27.29 ± 5.82	23.50 ± 7.14
	Single (134)	61.70 ± 24.52	25.64 ± 5.83	16.50 ± 5.27	24.94 ± 5.42	29.06 ± 6.28	25.35 ± 6.56

Statistics		*p*: 0.264 *t*: 1.120	**p** **: 0.033** *t*: 2.140	*p*: 0.482 *t*: −0.703	*p*: 0.273 *t*: −1.098	**p** **: 0.013** *t*: 2.503	**p** **: 0.023** *t*: 2.292

Having a child	Yes (133)	64.75 ± 20.52	24.16 ± 4.92	16.20 ± 4.59	24.10 ± 5.11	27.00 ± 5.71	23.16 ± 7.03
	No (159)	62.11 ± 23.69	25.51 ± 5.85	16.34 ± 5.15	24.97 ± 5.41	29.02 ± 6.26	25.35 ± 6.70

Statistics		*p*: 0.315 *t*: 1.007	**p** **: 0.036** *t*: 2.108	*p*: 0.804 *t*: −0.248	*p*: 0.162 *t*: −1.401	**p** **: 0.005** *t*: 2.851	**p** **: 0.007** *t*: 2.714

Educational status	High school and associate degree (45)	139.70	137.64	166.99	149.36	158.11	162.78
	Bachelor’s degree and graduate degree (247)	147.74	148.11	142.77	145.98	144.38	143.53

Statistics		*p*: 0.557 MWU: 5251.5	*p*: 0.443 MWU: 5159.0	*p*: 0.076 MWU: 4635.5	*p*: 0.805 MWU: 5429.0	*p*: 0.315 MWU: 5035.0	*p*: 0.158 MWU: 4825.0

Department studied^∗^	Internal medicine units (179)	154.92	143.03	153.78	145.44	146.41	147.90
	Surgical medicine units (113)	133.17	152.00	134.97	148.19	146.65	144.28

Statistics		**p** **: 0.032** MWU: 8607.0	*p*: 0.375 MWU: 9492.0	**p** **: 0.049** MWU: 8811.0	*p*: 0.786 MWU: 9923.0	*p*: 0.981 MWU: 10,096.0	*p*: 0.721 MWU: 9863.0

Working time^∗^	1–4 years (106)	159.83	160.16	149.42	149.87	160.93	162.10
	Over 5 years (186)	138.91	138.72	144.83	144.58	138.27	137.61

Statistics		**p** **: 0.042** MWU: 8445.5	**p** **: 0.036** MWU: 8410.5	*p*: 0.654 MWU: 9548.0	*p*: 0.605 MWU: 9500.5	**p** **: 0.027** MWU: 8328.0	**p** **: 0.017** MWU: 8204.5

^∗^ *Note:* *p* < 0.05; significance is shown in bold; mean rank means are given. t, Student T Test.

Abbreviations: MWU, Mann–Whitney *U* test; SD, standard deviation.

Participants who believed that their manager met their expectations, influenced their approach to care, communicated effectively, and adopted a team‐oriented approach reported significantly higher scores on the charismatic, reward, legitimate, and expert power subscales (*p* < 0.05). Conversely, coercive power scores were significantly higher among those who reported ineffective communication with their manager and perceived that their manager had no influence on their working conditions (*p* < 0.05) (Table 4).

**TABLE 4:** Comparison of nurses’ perceptions of their managers (NMPQ) with their scale scores.

NMPQ items	N:292	Reasons for nurses’ remaining silent total	Charismatic power	Coercive power	Rewarding power	Legal power	Expertise power
Manager’s fulfillment of expectations	Yes (116)	138.04	188.03	139.19	185.89	191.46	189.11
	No (176)	152.08	119.13	151.32	120.54	116.87	118.41

Statistics		*p*: 0.164 MWU: 9226.5	**p** < 0.001 MWU: 5390.0	*p*: 0.229 MWU: 9360.0	**p** < 0.001 MWU: 5638.5	**p** < 0.001 MWU: 4992.5	**p** < 0.001 MWU: 5265.0

To be able to communicate effectively with the manager	Yes (165)	147.12	172.08	137.52	169.48	170.62	167.41
	No (127)	145.69	113.27	158.17	116.64	115.17	119.33

Statistics		*p*: 0.885 MWU: 10,374.5	**p** < 0.001 MWU: 6257.5	**p** **: 0.038** MWU: 8995.5	**p** < 0.001 MWU: 6685.0	**p** < 0.001 MWU: 6498.5	**p** < 0.001 MWU: 7027.5

Manager’s influence on care behaviors	Yes (165)	153.55	154.05	140.05	154.59	157.46	155.65
	No (127)	134.66	133.83	157.32	132.92	128.09	131.14

Statistics		*p*: 0.064 MWU:8683.0	**p** **: 0.047** MWU: 8592.5	*p*: 0.090 MWU: 8794.0	**p** **: 0.033** MWU: 8493	**p** **: 0.004** MWU: 7962.0	**p** **: 0.016** MWU: 8299.5

Manager’s influence on working conditions	Yes (219)	148.82	147.97	136.73	151.73	149.08	148.98
	No (73)	139.55	142.10	175.82	130.81	138.76	139.05

Statistics		*p*: 0.417 MWU:7486.5	*p*: 0.064 MWU:7672.5	**p** **: 0.001** MWU:5853.5	*p*: 0.066 MWU:6848.0	*p*: 0.368 MWU:7428.0	*p*: 0.382 MWU:7449.0

The manager has a team approach	Yes (150)	150.57	177.44	139.46	174.14	176.98	175.90
	No (142)	142.20	113.82	153.94	117.30	114.30	115.44

Statistics		*p*: 0.397 MWU:10,040.0	**p** < 0.001 MWU:6009.5	*p*: 0.142 MWU:9593.5	**p** < 0.001 MWU:6503.5	**p** < 0.001 MWU:6077.5	**p** < 0.001 MWU:6240.5

*Note: p* < 0.05; significance is shown in bold; mean rank means are given.

Abbreviations: MWU, Mann–Whitney *U* test; SD, standard deviation.

In the study, a significant relationship was found between silence behavior and perceived coercive power in nurses (*p* < 0.05) (Table 5).

**TABLE 5:** The relationship between nurses’ silence and perceived power.

Reasons for nurses’ remaining silent total	Charismatic power	Coercive power	Rewarding power	Legal power	Expertise power
Pearson correlation	−0.007	0.336	−0.048	0.037	0.067
*p*	0.090	**0.001**	0.415	0.524	0.252

*Note:* Pearson correlation test; *p* < 0.05; significance is shown in bold.

## 4. Discussion

This study is significant because it is among the few to examine the relationship between nurse managers’ use of power and clinical nurses’ silence within the framework of organizational behavior in healthcare. It evaluates nurses’ silence not only in relation to individual and organizational factors but also as a phenomenon directly linked to how nurse managers exercise power. From the perspective of clinical nurses, this study contributes significantly to understanding management dynamics in healthcare and how a culture of silence is formed in the workplace. However, research on this topic remains scarce, with only a limited number of studies available in the literature.

### 4.1. Levels of Silence Behavior and Perceived Managerial Power Among Nurses

Nurses’ silence behavior was found to be below the scale midpoint. These findings are consistent with studies reporting moderate levels of organizational silence among nurses [18–20]. Moreover, nurses’ perceptions of their nurse managers’ use of charismatic, reward, legitimate, and expert power were above the scale midpoint, while their perception of coercive power fell below the midpoint. These results align with previous studies conducted in diverse healthcare settings. For instance, a study of intensive care nurses in Egypt found that head nurses frequently employed legitimate, expert, charismatic, and reward power, whereas their use of coercive power was low [21]. Similarly, a study of 230 nurses in a hospital ward in Saudi Arabia showed that nurses most frequently perceived their managers as using charismatic, legitimate, and expert power, while perceptions of coercive power were comparatively lower [22]. When nurses perceive their managers as relying on positive power sources such as charismatic, legitimate, and expert power, it may enhance their trust in leadership and their psychological safety. This suggests that nurses feel able to express themselves, leading to a decrease in silent behavior. Overall, these findings indicate that nurse managers across diverse cultural contexts tend to rely on positive power dynamics rather than coercive approaches, which may contribute to reduced professional silence among clinical nurses.

### 4.2. The Role of Sociodemographic Characteristics in Perceived Power and Silence Behavior

The findings indicate that single and childless participants perceived charismatic, expert, and legitimate power more strongly than their married or parenting counterparts. Although research on this topic is scarce, Akpınar similarly found that single nurses perceived their nurse managers’ legitimate power more highly [12]. These results suggest that nurses’ marital and parental status influence how they perceive the power of their nurse managers. According to the literature, leadership in organizations is shaped not only by leaders’ behaviors but also by employees’ individual characteristics and expectations [23]. In this context, single and childless nurses may devote more attention to their professional lives and adopt a more career‐oriented approach, as they have fewer family responsibilities. This greater focus on professional life may have enhanced their awareness of organizational roles and hierarchical structures, leading to higher perceptions of these power types. Furthermore, any interpretation of power perceptions must consider the differences between public and private hospitals in terms of organizational structure, hierarchy, perceived job security, and management practices. Additionally, the cross‐sectional design of the study and the absence of subgroup analysis by hospital type limit the ability to draw causal conclusions from these findings.

The study revealed that nurses in internal medicine units reported higher levels of coercive power perception and silence behavior compared to those in surgical units. Coercive power is defined as a manager’s ability to influence employee behavior through sanctions, punishment, or threats [23]. The higher patient turnover and ongoing nature of chronic care in internal medicine units may necessitate more frequent supervision, potentially leading managers to adopt a more authoritarian style and intensifying nurses’ perceptions of coercive power. Such an authoritarian approach may discourage nurses from voicing their opinions, resulting in increased silence behavior. Persistent silence among nurses can have adverse effects on both nurses and patients and may ultimately compromise organizational stability. This can lead to increased burnout, reduced work engagement, higher turnover intentions, and instability within the nursing team [24]. Therefore, it is essential for nurse managers to reassess their communication styles and embrace a more supportive, participative leadership approach. In internal medicine units, in particular, fostering open communication and creating a supportive environment where nurses feel safe to share their opinions could significantly reduce organizational silence and enhance job satisfaction.

Nurses with 1–4 years of experience reported higher perceptions of their nurse managers’ charismatic, legitimate, and expert power, as well as higher levels of employee silence, compared to those with 5 or more years of experience. Several factors may explain this finding: Less experienced nurses’ limited professional experience, combined with their manager’s hierarchical position and years of experience, may lead them to perceive their managers as having greater charismatic and expert power. This heightened perception of nurse managers’ expert and legitimate power may, in turn, contribute to increased silence among less experienced nurses. This silence may stem from several factors, including an inability to voice opinions openly, fear of being excluded, and a lack of professional experience.

Nurses who experience effective communication with their managers, feel that their expectations are met, and perceive a team‐oriented approach are more likely to attribute charismatic, reward, legitimate, and expert power to their managers. Conversely, those who struggle with communication and feel that their managers do not influence their working conditions tend to perceive their managers as relying on coercive power. These findings suggest that effective communication and transformational leadership enhance job satisfaction and reinforce positive power perceptions [25]. Supporting this, Salah et al. found that ward nurses who described their relationships with managers as “supportive,” “collaborative,” or “understanding” reported high job satisfaction, while those with negative relationships experienced dissatisfaction. The authors emphasized that the nurse–manager relationship is a key predictor of job satisfaction and influences the quality of patient care. Nurses in that study recommended that all nurse managers receive training in core leadership competencies, including effective communication, conflict resolution, and relationship‐building [26]. Similarly, Assi et al. reported that positive leadership traits fostered trust between nurses and managers and promoted a healthier work environment, leading to greater staff satisfaction and higher quality of care [27]. Tenza et al. further emphasized that nurse leaders’ focus on supportive leadership and organizational culture improvement is linked to better practice environments and care quality [28]. Collectively, these findings indicate that nurse managers’ leadership styles play a decisive role in shaping nurses’ perceptions of power. Nurse managers who adopt a supportive, team‐oriented approach and maintain open communication can enhance organizational climate and patient outcomes while cultivating positive power perceptions. In contrast, managers with poor communication skills, who discourage participation and rely on coercive power, may foster low motivation and job dissatisfaction.

### 4.3. The Relationship Between Nurse Managers’ Perceived Power and Clinical Nurses’ Silence Behavior

This study found a significant relationship between nurses’ silence and their perceptions of coercive power, indicating that authoritarian, oppressive, and punitive attitudes among nurse managers lead nurses to hesitate in expressing their thoughts. This finding aligns with literature on negative leadership and toxic leadership. For instance, Guo et al. reported that nurses characterized toxic leadership by excessive negative feedback, neglect, differential treatment, pressure on subordinates, self‐centeredness, and passivity. Most nurses in that study felt despair after encountering such leadership and chose to resign or transfer units [29]. Given that toxic leadership involves an oppressive and self‐centered style, it is closely linked to coercive power, driving nurses into silence and prompting them to consider leaving their positions. Similarly, Zou et al. found that acquiescent silence among nurses stemmed from negative leader behavior, fear of disrupting relationships, fear of horizontal violence, and fear of authority. They also noted that prolonged silence can adversely affect nurses and patients and threaten organizational stability [24]. Li et al. further demonstrated that negative leadership leads nurses to withhold suggestions and remain silent [30]. These findings suggest that an organizational climate suppressing employee voices weakens communication channels. The psychological burden of silence may result in burnout, job dissatisfaction, and turnover intention. Given the multifaceted nature of acquiescent silence, a comprehensive approach is needed to enhance organizational support for nurses. At the organizational level, fostering a fair and open communication environment and strengthening feedback mechanisms are essential. At the management level, cultivating trust, clarifying expectations, ensuring consistent messaging, and acknowledging nurses’ input are critical. Additionally, incentive systems should be established, and nurses should be empowered to participate in decision‐making.

Perceptions of power and preferences for participatory management are known to differ across cultural contexts [31]. Within Turkish culture, participatory management is relatively uncommon, and individuals often remain silent due to fear of or deference to authority figures [32]. Given that nurses’ silence is influenced by cultural norms and power distance as well as individual and managerial factors, it is essential to provide nurse managers with in‐service training on participatory leadership, open communication, and effective use of power. Furthermore, integrating rights‐based principles, advocacy skills, and critical thinking into nursing education can empower future nurses to develop a professional identity that actively counters organizational silence.

## 5. Conclusion

This study revealed significant relationships between nurses’ perceptions of managerial power types and their silence behaviors. Single and childless nurses reported higher perceptions of charismatic, expert, and legitimate power, whereas those in internal medicine units showed more pronounced silence and coercive power perceptions. Furthermore, nurses with 1–4 years of experience scored significantly higher on silence behaviors and perceptions of charismatic, legitimate, and expert power than those with five or more years in the profession. Nurses who felt their managers met expectations, communicated well, and used a team approach perceived significantly higher charismatic, reward, legitimate, and expert power. In contrast, those with ineffective communication and who believed their managers had no influence on their working conditions had higher coercive power perceptions. A significant relationship was found between perceived coercive power and nurses’ silence behavior. These findings indicate that nurses’ silence is closely linked to managers’ use of power, highlighting the critical role of leadership styles in organizational dynamics.

## 6. Strengths and Limitations of the Study

This study is one of the rare and original studies examining the relationships between clinical nurses’ perceptions of their managers’ use of power and their professional silence behaviors. Addressing a current and relatively understudied topic in the field of nursing management and organizational behavior constitutes one of the study’s significant strengths. Furthermore, the online and anonymized collection of data allowed participants to express their views more freely and increased the objectivity of the responses.

Nevertheless, several limitations of this study should be acknowledged. First, although the descriptive, relational, and cross‐sectional design enabled the examination of relationships between perceived managerial power and silence behavior, it precludes causal conclusions. Second, the use of self‐reported data may have introduced social desirability bias, and some participants may have withheld their true responses. Third, while the sample included nurses from both public and private institutions, potential differences between these sectors were not explored through detailed subgroup analysis. This restricts a full evaluation of contextual factors that could affect how power is perceived and how silence behaviors manifest. Additionally, employment status (e.g., temporary vs. permanent positions) was not evaluated, a factor that might have offered further understanding of silence behavior.

Furthermore, although the personal information form was developed based on relevant literature, reviewed by experts for content validity, and pilot‐tested prior to the main data collection, the second section of the form, hereinafter referred to as the NMPQ, which assesses clinical nurses’ perceptions of their nurse managers, did not undergo comprehensive psychometric validation procedures such as construct validity and reliability analyses. The use of this unvalidated measure may limit the strength of inferences drawn from these findings.

Finally, due to the scarcity of national studies on this topic, the discussion relied mainly on international literature, which limits the extent to which the findings can be interpreted within the local cultural context. Accordingly, the findings should be viewed with appropriate caution. Future research should employ designs that better reflect the national context, account for contextual variables, and include comprehensive validity and reliability analyses of newly developed measurement tools.

## Author Contributions

Conceptualization, data curation, formal analysis, funding acquisition, investigation, methodology, project administration, resources, software, supervision, validation, visualization, writing–original draft, and writing–review and editing: Şirin Çelikkanat, Ayşe Eminoğlu Güven, and Zeynep Güngörmüş.

## Funding

This research received no specific grant from funding agencies in the public, commercial, or not‐for‐profit sectors.

## Consent

Written informed consent was obtained from the nurses participating in the study.

## Conflicts of Interest

The authors declare no conflicts of interest.

## Data Availability

Anonymous data supporting the findings of this study can be obtained from the relevant author upon reasonable request.

## Appendix 1

*Appendix A1: Personal Information Form*.

1. Age:
2. Gender: a. Male b. Female
3. What is your marital status? a. Married b. Single
4. Do you have children? a. Yes b. No
5. What is your educational background?
  a. High school and associate degree b. Bachelor’s and graduate degree
6. In which department do you work? a. Internal medicine department b. Surgical department
7. How long have you been working as a nurse? a. 1–4 years b. 5 years or more

*Appendix A2: Nurse Manager Perception Questionnaire*.

8. Do your managers meet your expectations regarding your profession?
  a. Yes b. No
9. Are you able to communicate effectively with your managers?
  a. Yes b. No
10. Do you think your managers influence your care practices?
  a. Yes b. No
11. Do you think your managers influence your working conditions?
  a. Yes b. No
12. Do you think your managers have a team‐oriented approach?
  a. Yes b. No
